# Microcrystal electron diffraction-guided discovery of natural products

**DOI:** 10.1063/4.0001091

**Published:** 2025-10-27

**Authors:** David A Delgadillo

**Affiliations:** California Institute of Technology, Pasadena, CA, USA

## Abstract

Nature remains a vast repository of complex and functional metabolites whose structural characterization continues to drive innovations in pharmaceuticals, agrochemicals, and materials science. The cryogenic electron microscopy (cryoEM) method, microcrystal electron diffraction (microED, a 3D ED technique) has emerged as a powerful tool to structurally characterize small molecules. Despite this emerging role in structural chemistry, the cost and throughput of microED have limited its application in the discovery of natural products (NPs). While recent advances in sample preparation (e.g. ArrayED) have provided a conceptual framework to address these challenges, they have remained unproven. Herein, we report the ArrayED-driven discovery of a structurally-unprecedented family of NPs (zopalide A-E), a muurolane-type sesquiterpene glycoside (rhytidoside A), aspergillicin analogs (aspergillicin H and aspergillicin I), and four crystal structures of previously reported fungal metabolites. We provide the first examples of absolute stereochemistry determination *via* microED for newly annotated NPs. Moreover, we cover cases where careful analysis ab initio solutions can direct the need for further resolution of structurally similar NPs.

